# Novel Liposome–Gel Formulations Containing a Next Generation Postbiotic: Characterization, Rheological, Stability, Release Kinetic, and In Vitro Antimicrobial Activity Studies

**DOI:** 10.3390/gels10110746

**Published:** 2024-11-15

**Authors:** Halise Betül Gökçe, İsmail Aslan

**Affiliations:** 1 Department of Pharmaceutical Technology, Faculty of Pharmacy, Afyonkarahisar Health Sciences University, Afyonkarahisar 03030, Turkey; 2 Department of Pharmaceutical Technology, Hamidiye Faculty of Pharmacy, University of Health Sciences, Istanbul 34668, Turkey; eczismailaslan@gmail.com

**Keywords:** liposomes, gels, lipogelosomes, drug delivery systems, postbiotics, antimicrobial effect

## Abstract

In recent years, in addition to the positive effects of probiotics and prebiotics on health, increasing research has shown that postbiotics also have significant potential in the health field. Postbiotics are bioactive components produced by probiotic bacteria during fermentation and may exhibit antimicrobial activity. This study investigated the antimicrobial effects of liposomal postbiotics formulated in gel. Various postbiotic-containing liposomal systems have been developed and optimized to prepare formulations. Optimized liposomes and liposomal postbiotic-containing gel forms were examined in terms of particle size, polydispersity index, zeta potential, structural properties, encapsulation efficiency, permeability, release profiles, and stability. Finally, the antimicrobial activities of the postbiotics and the optimum gel formulation LG1 were evaluated on *Pseudomonas aeruginosa*, *Staphylococcus aureus*, *Escherichia coli*, *Enterococcus hirae*, and *Candida albicans* strains using disk diffusion and microdilution methods. The optimum liposome formulation L1 was determined to have a particle size of 185.32 ± 0.80 nm, a polydispersity index of 0.206 ± 0.012, a zeta potential of 35.0 ± 0.5 mV, and an encapsulation efficiency of 17.52%. Its permeability was determined as 51.52% at the end of 6 h. In vitro release studies showed that the drug release profile was in accordance with first-order kinetics and suitable for controlled release. The findings show that formulated postbiotics have similar antimicrobial activity to free postbiotics. These results suggest that liposomal gel formulations support the antimicrobial effects of postbiotics while providing advantages of use. In conclusion, the findings contribute to a better understanding of the antimicrobial potential of postbiotics and lipogelosomal postbiotics and optimize their use in pharmaceutical applications.

## 1. Introduction

The microbiome refers to the diverse community of microorganisms that usually live inside the human or animal body or on the skin. Many commensal, symbiotic, and pathogenic microorganisms form a complex ecosystem on the organism [1]. The microbiota formed by these microorganisms can exert various effects on health through probiotics, prebiotics, and, more recently, postbiotics [2].

Probiotics are living microorganisms that provide health benefits to the host when administered in adequate amounts. The term “probiotics” is used to describe a group of bacterial species, including lactobacilli, bifidobacteria, and yeast, which have been demonstrated to confer benefits to human health and interact synergistically with human physiology. Probiotics have been shown to play a direct role in maintaining a “healthy” immune system. Additionally, they have been shown to prevent the dysbiosis of the gut microbiota that occurs after antibiotic use and to combat opportunistic pathogens [3,4]. Prebiotics are indigestible food components used for the nutrition of probiotics. Postbiotics are defined as bioactive components produced by probiotics during their life cycle. These components provide various benefits to the organism [2,3,4,5,6]. In recent years, much research has been conducted focusing on the antimicrobial activity of postbiotics. Postbiotics have been shown to have inhibitory effects against various pathogenic microorganisms. This highlights the potential for postbiotics, such as *Lactobacillus* lysates, to have antibacterial, antifungal, and antiviral properties [7,8,9,10,11]. However, they do not directly contain live microorganisms and are more stable, which makes postbiotics advantageous over probiotics [2,3,4,5,6].

Among the various drug delivery systems, liposomes represent an optimal choice for enhancing the effects of postbiotics. The FDA defines liposomes as vesicles composed of single (unilamellar) or multiple (multilamellar) bilayers formed from amphipathic molecules such as phospholipids, separated by aqueous compartments surrounding a central aqueous core. Liposomes have many advantages compared to conventional drug delivery systems, including targeting potential, sustained or controlled release, protection of drugs against degradation, superior therapeutic effects, lower toxicity, and reduced side effects. In this respect, liposomes have been recognized as promising and versatile drug vesicles [12,13].

While liposomes have significant potential to deliver active ingredients to skin structures, liposomes in hydrogel formulation have been reported to be more suitable for topical application than other semi-solid dosage forms, including ointments and creams. Novel carrier system strategies have been studied in the literature to develop suitable dosage forms of liposomes for dermal delivery. Among these strategies, hydrogels stand out for their versatility and advantages. Hydrogels are widely used in the food and pharmaceutical industries due to their biodegradability and biocompatibility. As a result, the suitability of topical gel forms designed to carry liposomes for dermal applications is mentioned [14,15,16,17].

In this study, first, liposome and lipogelosome (liposome in hydrogel) formulations containing postbiotics will be developed and optimized by characterization studies. Then, the data obtained using the disk diffusion method and microdilution will be analyzed to evaluate the antimicrobial effects of the formulations for which stability studies were performed. This study will provide a better understanding of the antimicrobial properties of postbiotics and provide data that will form the basis for future pharmaceutical applications.

This study aimed to investigate the antimicrobial activity of postbiotics and an optimally formulated liposomal gel. The results confirm that postbiotics alone have antimicrobial activity, while the liposomal formulations developed enhance this activity by providing ease of use.

## 2. Results and Discussion

### 2.1. Particle Size, Polydispersity Index, and Zeta Potential

Values obtained from characterization studies on liposome dispersions are given in Table 1 (n = 6).

### 2.2. Rheological Behavior

**Figure 1:**
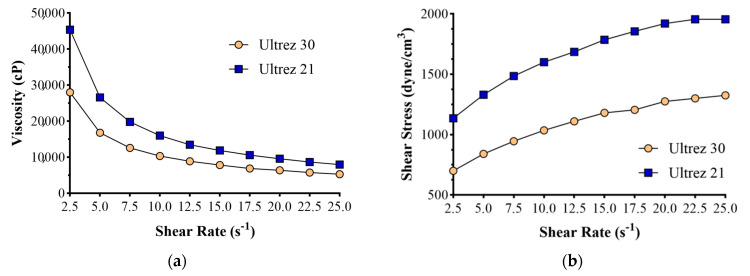
Graphs of viscosity (**a**) and shear stress (**b**) versus shear rate of LG1 (containing Ultrez 21) and LG4 (containing Ultrez 30) coded formulations.

The analysis measured the viscosity and shear stress of LG1 and LG4 gel formulations obtained by formulating postbiotic and L1 formulations with U-21 (Ultrez-21) and U-30 (Ultrez-30) gelling agents against increasing shear rates, and their rheological behaviors were evaluated. Graphs of viscosity and shear stress versus shear rate for both gels are shown in Figure 1.

Since the LG1 formulation’s shear stress values are higher than LG4’s, the LG1 formulation’s stability is also expected to be higher. Sample LG4 may flow at a lower shear rate than LG1. Since topical applications require a specific contact time between the formulation and the skin, it is advantageous for gel formulations to stay longer, even at a higher shear rate. This shows that the LG1 gel prepared with Ultrez 21 will provide a usage advantage.

### 2.3. FT-IR

**Figure 2:**
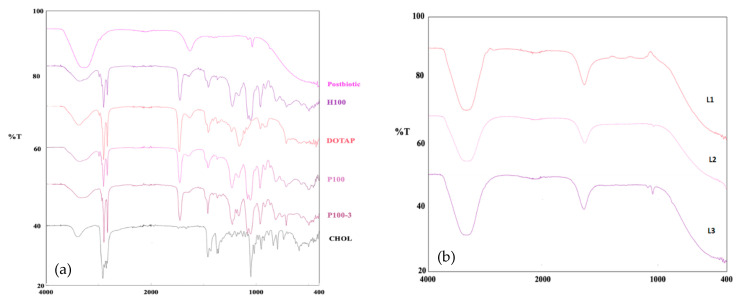
(**a**) FT-IR spectra of raw materials used in liposomes; (**b**) FT-IR spectra of liposomes.

FT-IR spectra of the liposome formulations and formulation components were analyzed. As a result of the examination, a broad band is observed in the 3000–3600 cm^−1^ range. This band is thought to consist of the OH bond in the structure of the cholesterol molecule and the characteristic band obtained as a result of FT-IR analysis of the postbiotic. The band in the 1600–1700 cm^−1^ range is thought to be due to the C=C structure, presumably from DOTAP (1,2-dioleoyl-3-trimethylammonium propane), CHOL (Cholesterol), and postbiotics. The spectra and bands are summarized in Figure 2 [18,19,20,21,22].

When the postbiotic FT-IR spectrum is examined, a signal is seen at approximately 1050 cm^−1^. This signal is thought to come from sulfoxide and primary alcohol. The signal seen at approximately 1700 cm^−1^ is a slightly broad and not a sharp peak. This peak is thought to belong to the carbonyl group and, in particular, it is thought to be a carboxylic acid. A band belonging to the OH group is seen in the range of 3500–4000 cm^−1^ [18,19,20,21,22].

For the detailed examination of the L2 formulation, the characteristic signal at approximately 1050 cm^−1^ in postbiotics is seen here. The signals belonging to cholesterol, DOTAP, and P100-3 appearing at approximately 1700 cm^−1^ are also seen in this formulation. Since the peaks generally appear in the same places, there is overlap and it is seen that all three peaks appear in the same place.

### 2.4. Differential Scanning Calorimetry (DSC)

**Figure 3:**
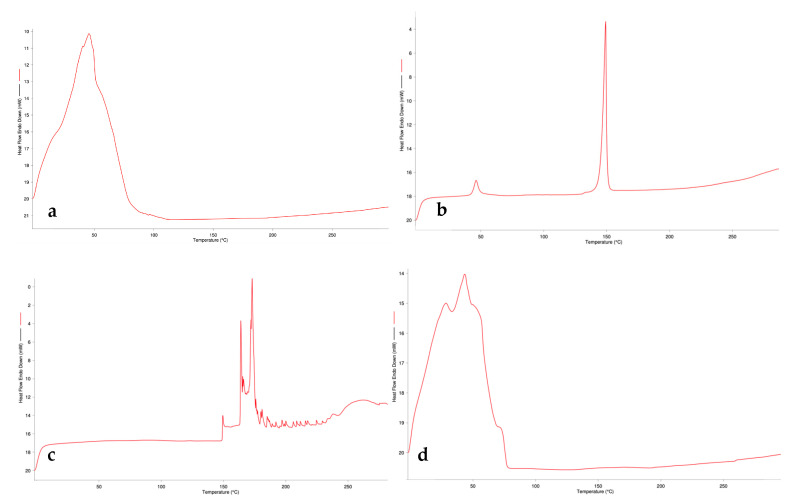

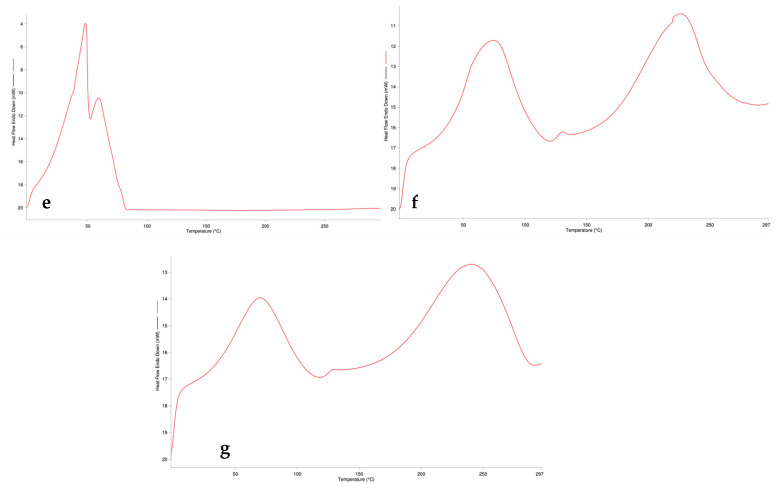
DSC thermograms of raw materials: (**a**) Postbiotic; (**b**) Cholesterol; (**c**) H100; (**d**) P100; (**e**) P100-3; (**f**) Ultrez30; (**g**) Ultrez21.

**Figure 4:**
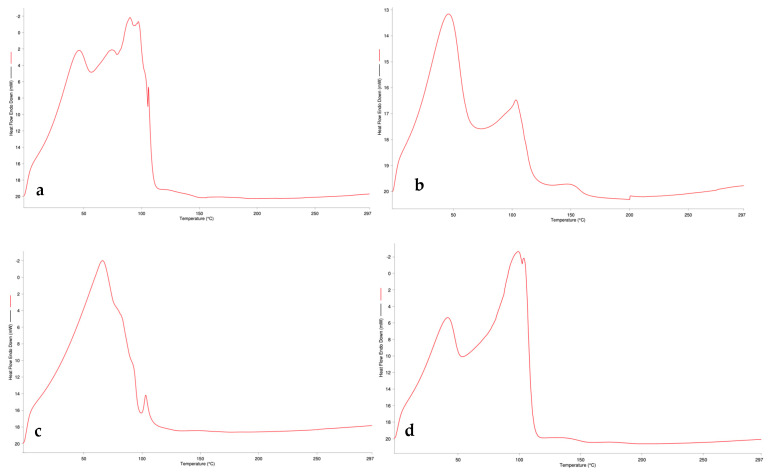

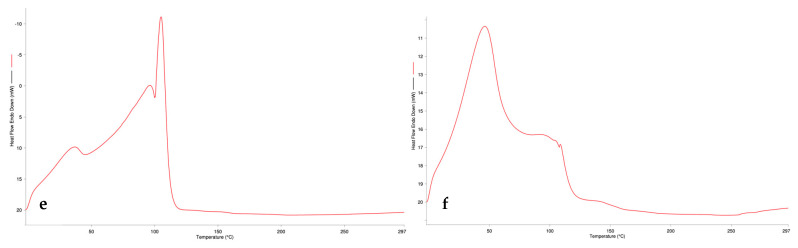
DSC thermograms of the formulations: (**a**) LG1; (**b**) LG2; (**c**) LG3; (**d**) LG4; (**e**) LG5; (**f**) LG6.

Thermograms were obtained between 0 and 300 °C at a temperature increase of 10 °C/min and a nitrogen flow rate of 20 mL/min. The thermograms of the raw materials used are shown in Figure 3, and the thermograms of the formulations are shown in Figure 4.

The physical properties, purity, and interactions of the raw materials with the excipients were evaluated by DSC thermal analysis [23]. U-30 and U-21 gelling polymers gave similar bands at approximately 60 °C and 230 °C. The similarity of their chemical structures explains the similarity of the graphs. P100 and P100-3 phospholipids, which represent different modifications of soy phosphatidylcholine, also show similar bands for this reason. When the thermograms of the formulations are analyzed, it can be stated that the bands are spread in the range of 0–100 °C, and no sharp band is observed after 150 °C.

### 2.5. Encapsulation Efficiency

The HPLC method determined that the L1, L2, and L3 formulation had 12.5, 11.1, and 8.6 ppm lactic acid from the liposome formulation containing postbiotics, respectively. Equation (1) calculated the encapsulation efficiencies as 17.52%, 15.56%, and 12.05%.

**Figure 5:**
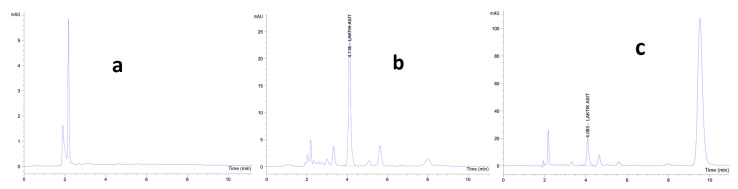
HPLC chromatograms. (**a**) Mobile phase; (**b**) Lactic acid containing postbiotic sample; (**c**) Postbiotic lysate-containing liposome sample.

Evaluating these results reveals that the more effective drug loading capacity of the L1 formulation is critical in deciding on the optimum drug formulation. Figure 5 presents the HPLC chromatograms of the mobile phase, postbiotic lysate, and liposomal system, which have been employed for the comparison of the sample analyzed in the study with reference lactic acid standard.

### 2.6. In Vitro Release Studies

**Figure 6:**
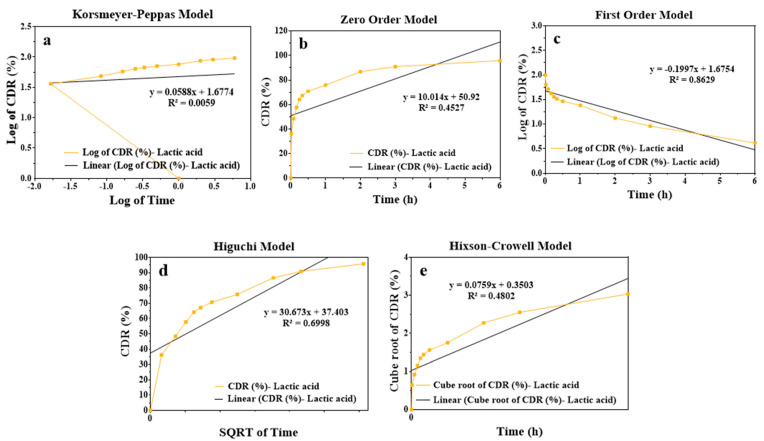
Graphs of (**a**) Korsmeyer–Peppas; (**b**) Zero order; (**c**) First order; (**d**) Higuchi; (**e**) Hixson–Crowell kinetic models. CDR: Cumulative drug release quantity, SQRTT: square root of time.

Figure 6 shows graphs of Korsmeyer–Peppas, zero-order, first-order, Higuchi, and Hixson–Crowell kinetic models generated with data obtained from drug release studies.

When time is plotted on the *X*-axis and the cumulative logarithmic percentage of the drug on the *Y*-axis, the line obtained gives an idea about the conformity of the drug release to first-order kinetics. Considering the R^2^ values, the release kinetics fitting the LG1 formulation is a first-order model [24].

In this case, the release rate of the prepared drug carrier system depends on the drug concentration. According to this kinetic model, the drug concentration is proportional to the release rate. This indicates that when the drug concentration is high, the release rate will be high, and when the drug concentration decreases, the release rate will also decrease [20,24].

### 2.7. In Vitro Permeation Studies

**Figure 7:**
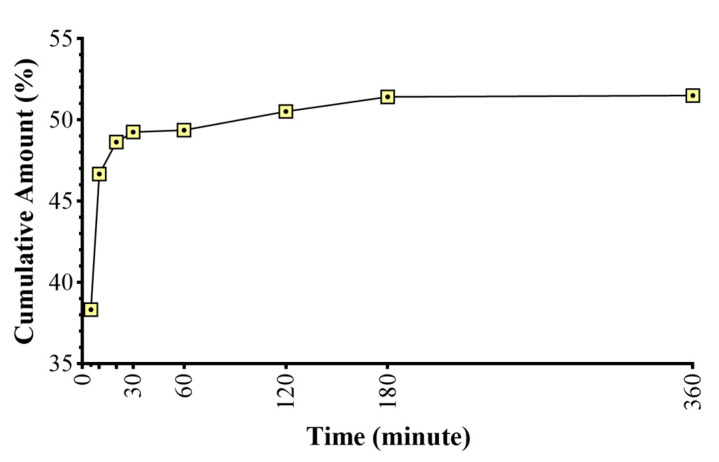
Graph of % cumulative lactic acid content versus time.

The results of the measurements made from the samples taken at 1, 5, 10, 20, 30, 30, 60, 120, 180, and 360 min are shown in Figure 7 as % cumulative lactic acid content versus time. As can be seen from the graph, there is a rapid increase in the permeation until the 10th minute. However, there is no similar increase in the permeation amount, especially after the 1st hour. After considering the dilution factors, the permeability was calculated as 51.52% at the end of the 6th hour.

### 2.8. Disk Diffusion Method

The disk diffusion method is one of the most widely used methods for evaluating postbiotic antimicrobial activity. This simple, rapid, and economical method can be used to determine antimicrobial activity. The zone of inhibition formed around these agents is determined by measuring the rate of diffusion of antimicrobial agents in a matrix. Evaluation of postbiotics by disk diffusion is a widely used method to determine the antimicrobial potential of these compounds [25,26,27,28,29]. However, since it is not possible to measure the amount of the antimicrobial agent diffusing into the agar medium with the disk diffusion method, it is not suitable for determining the minimum inhibitory concentration (MIC). Therefore, MIC values will be determined by the microdilution method [26,30].

The study was performed as described in Section 4.10. The resulting inhibition diameter (mm) values are summarized in Table 2.

The antimicrobial activities of liposomal postbiotics and lipogelosomal formulation postbiotics were investigated using the agar disk diffusion method. When the zone diameters were analyzed, it was observed that *C. albicans* was the least susceptible microorganism, but a significant antimicrobial effect was observed on all strains. When postbiotic and lipogelosomal postbiotic formulations were compared, it was observed that the formulations did not contribute positively to the antimicrobial effect in all strains except for *E. coli*, but no significant decrease was observed.

### 2.9. Microdilution Method

The minimum inhibitory concentration (MIC) values of postbiotic and liposomal gel formulations determined according to the microdilution method are given in Table 3.

The study showed that postbiotics formulated in liposomal gel may have antibacterial activity at lower concentrations. When the MIC results were analyzed, it was observed that the samples showed higher activity in *P. aeruginosa* and *C. albicans* strains with a concentration of 1.25% compared to other strains. In addition, *S. aureus* and *E. coli* strains showed less sensitivity than others.

### 2.10. Stability Studies

The Turbiscan Stability Index (TSI) value, a device-specific index, indicates the increasing destabilization of the product. The company states that the product has a visible stability problem, especially with TSI values exceeding 10. In addition, the maximum TSI value of the product at the end of the analysis is also a guide for comparing the stability between products.

Table 4 shows the critical TSI values of 3 and 10 and the TSI values at the end of 24 h. In addition, the TSI values at the end of 24 h for L1, L2, and L3 formulations were determined as 14.5, 24, and 31. Considering these values, the L1 formulation will show a more stable profile than the others.

**Figure 8:**
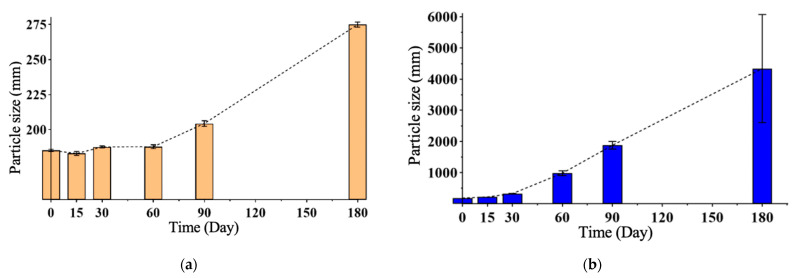
(**a**) Particle size versus time at 4 ± 2 °C and 60 ± 5% RH. (**b**) Particle size versus time at 25 ± 2 °C and 60 ± 5% RH.

**Figure 9:**
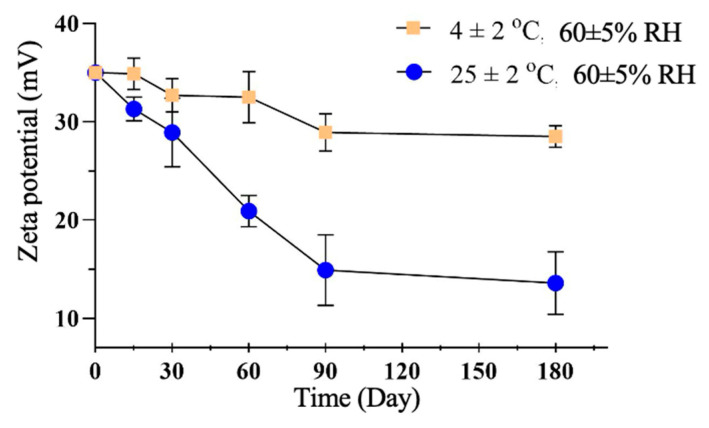
Zeta potential versus time at 4 ± 2 °C and 25 ± 2 °C and 60 ± 5% RH.

After the studies, it was predicted that the L1 formulation would be more stable in the long term, and real-time physical stability studies were initiated with the L1 formulation. The data of the study carried out for 180 days at 4 ± 2 °C and 25 ± 2 °C in a 60 ± 5% relative humidity environment for the L1 formulation are given in Table 5 and Table 6, and the studies are summarized in Figure 8 and Figure 9.

In the long-term stability studies on the LG1 formulation, particle size, PDI, and zeta potential measurements show that it is more stable at 4 ± 2 °C temperature and 60 ± 5% relative humidity.

Postbiotics derived from probiotic bacteria have recently attracted the close attention of researchers due to their remarkable effects and minimal adverse effects. Although these compounds are natural, they are claimed to pose no risk of bacteremia for immunocompromised individuals. They, therefore, have great potential for industrial applications related to food and pharmaceutical products [7,8,9,10,11].

## 3. Conclusions

When the prepared formulations are examined, it is seen that they have a particle size around 200 nm and show cationic properties. DOTAP phospholipids in the formulation components explain the cationic charge of the formulations. The cationic nature of liposomes positively affects the penetration capacity of drug active ingredients into the skin by showing high affinity for the anionic skin membrane. However, cationic liposomes have the advantage of higher physical stability and encapsulation efficiency due to their surface charge [31,32,33].

As a result of the encapsulation efficiency and preliminary stability studies on the three formulations, optimization studies were carried out on LG1 and LG4 formulations due to the high encapsulation capacity of the L1 formulation compared to the other.

Figure 1 shows that both LG1 and LG4 formulations showed pseudoplastic flow. The flow behavior of a formulation before and after application can be inferred from its shear stress value at a low shear rate. In topical formulations, it is desirable that the formulation shows decreasing viscosity with increasing shear rate. In order for the formulation to be easily removed from the package, it should not flow when the shear rate is low, that is, it should show a resistance to flow. Similarly, it is expected to spread to the skin as the shear rate increases during application. Since a certain contact time is required between the formulation and the skin in topical applications, it is an advantage for gel formulations that the formulation can stay longer even at higher shear rate values [34].

According to the release kinetics study on LG1 formulation, the designed gel form drug carrier system releases in accordance with the first-order release kinetics where the release depends on the drug concentration [19,24].

The antimicrobial activity of the postbiotic and liposomal gel formulations was investigated qualitatively and quantitatively based on disk diffusion and microdilution experiments, evaluating the presence of inhibition zones, zone diameters, and MIC values. In disk diffusion experiments, antimicrobial activity was detected with the presence of zones on all microorganisms examined (Table 2). It should be noted that the samples showed different antimicrobial activity against the tested microorganisms.

According to the results of the disk diffusion test, the diameters of the inhibition zones of liposomal postbiotics on *Escherichia coli*, *Staphylococcus aureus*, and *Candida albicans* strains were found to be larger than those of postbiotics. In particular, the diameters of inhibition zones on *Staphylococcus aureus* strains were increased when liposomal postbiotics were used.

These results suggest that liposomal formulation can enhance the antimicrobial activity of postbiotics. This study supports using postbiotics and lipogelosomal postbiotics as potential antimicrobial agents in pharmaceutical applications. Due to their antibacterial and antifungal effects, postbiotics and liposomal formulations may be considered an alternative to antibiotics.

In conclusion, this study contributes to a better understanding of the antimicrobial effects of postbiotics and liposomal postbiotics. Further research is needed to utilize postbiotics and liposomal formulations as alternative antimicrobial agents in pharmaceutical applications. The results of this study may form the basis for further research in this direction.

## 4. Materials and Methods

### 4.1. Materials

All phospholipids, phosphatidylcholine from non-GMO sunflowers (H100), phosphatidylcholine from non-GMO soybeans (P100), hydrogenated phosphatidylcholine from non-GMO soybeans (P100-3), and 1,2-dioleoyloxy-3-trimethylammonium propane chloride (DOTAP) were purchased from Lipoid AG (Ludwigshafen, Germany). Cholesterol (CHOL), Triton X-100, Mueller–Hinton Broth, and Mueller–Hinton Agar were purchased from Sigma Chemical Co. (St. Louis, MO, USA). Sabouraud Dextrose Agar, Sabouraud Dextrose Broth, Sabouraud Dextrose chloramphenicol Agar, and Potato Dextrose Agar were purchased from Neogen (Lansing, MI, USA). Ultrez 30 (U-30) and Ultrez 21 (U-21) were purchased from Lubrizol (Noveon) (Milan, Italy).

The postbiotic used in the study was graciously provided by SFA Research and Development & Private Health Services and ATABIO Technology Ltd., Co., (Istanbul, Turkiye). The active ingredients are a postbiotic (ATA-LTW 35), coconut fruit extract, as well as a combination of the *Lactobacillus acidophilus* (ATA-LAP1201), *Lactobacillus reuterii* (ATA-LTC-Lr050600), *Bifidobacterium animalis* (ATA-BSLA0310), and *Streptococcus thermophilus* (ATA-LTC St140700), which have undergone fermentation. *Pseudomonas aeruginosa* (ATCC 9027), *Candida albicans* (ATCC 10231), *Staphylococcus aureus* (ATCC 6538), *Escherichia coli* (ATCC 8739), and *Enterococcus hirae* (ATCC 10541) were used as pathogen microorganisms.

All other materials were analytical grade and used without purification.

### 4.2. Preparation of Liposome Dispersions and Liposomal Gel Formulations

The thin film lipid layer method was used to prepare liposome dispersions. The extrusion method was used to achieve the desired particle size [31,32]. Lipid dispersions were prepared by dissolving 20 mg of lipid with a 2:1 molar ratio of chloroform/methanol [33]. Three different liposome formulations were designed. The formulations and their codes are shown in Table 7.

The organic solvent was removed using a rotary evaporator (Heidolph, Germany) under reduced pressure at 150–200 rpm and a temperature of 30–40 °C. The thin film layer obtained by evaporation of the organic solvent was hydrated with ultrapure water. The hydration process was carried out with 1 mL of ultrapure water for every 1 mg of lipid at a temperature of 40 ± 2 °C, which was determined by considering the lipid’s phase transition temperature. The postbiotic (20%) was encapsulated using the passive encapsulation method during the hydration process. Meanwhile, inert glass spheres with a diameter not exceeding 2 mm separated the thin film from the glass surface and formed multilayer liposomes [32,34,35].

The extrusion method was used to obtain monodisperse liposomes. In this process, the liposome dispersion was monodispersed by passing through polycarbonate filters, allowing liposomal vesicles to form in the desired size range and homogeneity. The extrusion process was repeated 4 times using a 400 nm and 200 nm polycarbonate membrane filter, respectively [32,36,37].

Liposome–gel formulations were prepared by incorporating postbiotic liposomes into gel carriers. Due to their good bioadhesive properties, the carbopol derivatives Ultrez 21 (U-21) and Ultrez 30 (U-30) were used as gelling agents at 0.5% concentration in distilled water [14,38]. The composition of the prepared formulation is given in Table 8.

Six different gel formulations were designed. All formulations are shown in Table 9.

### 4.3. Particle Size, Polydispersity Index, and Zeta Potential

The dynamic light scattering method measured the liposome dispersions’ average particle size and particle size distributions. The zeta potential values of the developed formulations were measured using the electrophoretic light scattering method (Malvern Instruments Mastersizer 2000, Worcestershire, UK). The formulations were diluted with ultrapure water and analyzed (n = 3) [39,40,41].

### 4.4. Rheological Behavior

Viscosity measurements of LG1 and LG4 lipogelosomes were performed at 25 °C using a rheometer (Brookfield DV3T, Middleboro, MA, USA). Viscosity and shear stresses were recorded at increasing shear rates. The rheological behavior of the formulations was interpreted by obtaining viscosity and shear stress curves for different shear rate values [42].

### 4.5. FT-IR

The FT-IR spectrum of formulations and raw materials was scanned across the wavenumber range of 400–4000 cm⁻^1^ (Thermo Fisher Scientific, Cleveland, OH, USA) [43].

### 4.6. Differential Scanning Calorimetry (DSC)

The DSC method was used for the thermal analysis of the formulations. Samples were taken in aluminum pans and evaluated at a temperature increase of 10 °C/min between 0 and 300 °C, at a nitrogen flow rate of 20 mL/min, and thermograms were obtained. Empty aluminum pans were used as references [44,45].

### 4.7. Encapsulation Efficiency

Liposomal drug carrier systems containing postbiotics, coded L1, L2, and L3, were prepared as described in Section 4.2. The encapsulation efficiencies of the three liposomal drug carrier systems were determined and compared.

In order to ascertain the proportion of the active substance incorporated into liposome dispersions, ultracentrifugation was conducted at 15,000 rpm for 45 min at 4 °C. After adding 5% Triton X-100 to the precipitate and diluting with the mobile phase, the precipitate was passed through a regenerated cellulose filter and determined by HPLC. The lactic acid (LA) metabolite in the postbiotic was monitored in the HPLC analysis. The encapsulation efficiency was calculated using Equation (1) [46,47]:

(1) $$\mathrm{Encapsulation}\;\mathrm{Efficiency}\%=\frac{The\;amount\;of\;LA\;obtained\;as\;a\;result\;of\;the\;analysis\;\times 100}{Amount\;of\;LA\;added}$$

### 4.8. In Vitro Release Study

The release of postbiotics from lipogelosome formulations was investigated using the dialysis membrane method. Postbiotic release from formulations in cellulose dialysis membranes was carried out in 200 mL of ultrapure water at 37 °C. At certain time intervals (1, 5, 10, 15, 20, 30, 45, 60, 120, 180, and 360 min), 1 mL of the sample was removed and replaced with the same fresh medium. The lactic acid amounts in the samples were analyzed using HPLC [42].

With the results obtained, the drug release kinetics were analyzed by mathematical methods per zero-order, first-order, Higuchi, Korsmeyer–Peppas, and Hixson–Crowell kinetics. The equations of these kinetics are given in Table 10 [25,30,48,49].

Q is the fraction of drug released at time interval t; k, k_0_, k_1_, k_h_, and k_hc_ are the values of the kinetic constants related to Krosmeyer–Peppas, zero-order, first-order, Higuchi, and Hixson–Crowell models, respectively; and n is the diffusion exponent, accounting for the mechanism of drug release.

### 4.9. In Vitro Permeation Studies

Permeation studies with Franz diffusion cells were performed using polydimethylsiloxane (PDMS) membranes, a simplified human epidermis model. PDMS membranes are widely used in pre-permeation studies due to their simple and reproducible structure. The LG1 formulation was placed in the donor compartment of the diffusion cells. The effective diffusion area is 0.95 cm^2^, and the average volume of the receptor chamber is approximately 3.9 mL [50,51].

The receptor chamber was filled with ultrapure water and continuously stirred at 350 rpm. The receptor chambers of the diffusion cells were kept at 37 °C using a recirculating water bath. For quantification by HPLC, 1 mL of the sample was removed at the designated times (1, 5, 10, 20, 30, 60, 120, 180, and 360 min) and immediately replaced with an equal volume of fresh medium [50].

### 4.10. Disk Diffusion Method

The disk diffusion method used strains of *Pseudomonas aeruginosa* (ATCC 9027), *Candida albicans* (ATCC 10231), *Staphylococcus aureus* (ATCC 6538), *Escherichia coli* (ATCC 8739), and *Enterococcus hirae* (ATCC 10541). The study was carried out on postbiotic lysate and postbiotic-loaded liposomal gel formulations.

Subcultures removed from the freezer were streaked on Triptin Soy Agar (TSA) and Sabouroud Dextrose Chloramphenicol Agar (SDA). Bacteria were incubated at 32.5 ± 2.5 °C for 18–24 h and yeasts at 22 ± 2.5 °C. Microorganisms were removed from the subculture with the help of a pipette and diluted with peptone salt solution. The homogenous suspension was inoculated on TSA, SDA, and Potato Dextrose Agar (PDA) media, and the number of microorganisms was determined. Microorganism suspensions of 10^7^ cfu/mL for bacteria and 10^5^–10^6^ cfu/mL for yeasts were prepared in the McFarland apparatus. The suspensions were spread on the TSA, SDA, and PDA surfaces [52,53,54,55,56,57].

A total of 15 µL of the sample was impregnated on a 6 mm diameter blank disk and placed on the agar surface. Ofloxacin (5 µg/disk) and the reference standard nystatin (100 IU) were positive controls for each microbial species tested. A blank disk impregnated with buffer was used as a negative control. Inoculated plates were incubated at 32.5 ± 2.5 °C for 18–24 h for bacteria and 22 ± 2.5 °C for yeasts. The inhibition diameter of the organism tested for antimicrobial activity was measured with a millimeter ruler, and the area of inhibition was calculated [52,54,55].

### 4.11. Microdilution Method

The microdilution method determined the minimum inhibitory concentration, as recommended by the Clinical and Laboratory Standards Institute. *Pseudomonas aeruginosa* (ATCC 9027), *Staphylococcus aureus* (ATCC 6538), *Escherichia coli* (ATCC 8739), and *Candida albicans* (ATCC 10231) strains were used in this method. The study was carried out on postbiotic lysate and postbiotic-loaded liposomal gel formulations.

A total of 100 μL of cation-adjusted Mueller–Hinton Broth (MHB) for bacteria and Sabouraud Dextrose Broth (SDB) for yeast was added to all wells of 96-well plates. Each row of 12 wells of the plates was used for a single bacterial strain. The first well was left blank to monitor growth control. The samples were serially diluted through 5 wells [58,59].

One day before the study, all microorganisms were inoculated on Mueller–Hinton Agar and Sabouraud Dextrose Agar medium in 96-well plates and incubated at 36 °C for 24 h and 27 ± 1 °C for 48–72 h. The next day, the purity of the microorganisms was checked. The microorganism suspension adjusted to a McFarland turbidity of 0.5 was diluted 1/100 with MHB and SDB, and 100 μL was inoculated into each well with a final concentration of 5 × 10^5^ [60,61].

The plate lid was closed, shaken at 300 rpm, and left to incubate. Incubation was performed at 36 ± 1 °C for 18–24 h for bacteria and at 27 ± 1 °C for 48 and 72 h for yeast. At the end of incubation, microbial growth was determined by measuring the absorbance in each well on a plate reader. Absorbance was read at a wavelength of 600 nm for bacteria and 530 nm for yeast using a microplate reader (Biotek Instrument Inc., Highland Park, VT, USA). The last well without growth was considered as the MIC value [60,61].

### 4.12. Stability Studies

In order to examine the physical stability of the liposome formulations, a pre-stability test and a 6-month physical stability test were performed. In the pre-stability test, Turbiscan (Formulaction, Toulouse, France) was used for the investigation and early detection of physical stability problems in L1, L2, and L3 formulations and for optimizing the formulation. Samples were taken in a 20 mL glass bottle with a height of 42 mm. The analysis was continued for 1 day at 30 ± 2 °C, and the signal value was recorded every 60 min [62,63,64].

After Turbiscan analysis, the selected liposome dispersion’s formulation (L1) was analyzed for 6 months at two different temperature environments, 4 ± 2 °C and 25 ± 2 °C at 60 ± 5% relative humidity (RH). Particle size, polydispersity index, and zeta potential measurements were performed at 0, 15, 30, 60, 90, and 180 days (n = 6) [65,66,67].

### 4.13. Statistical Analysis

Statistical analysis and data visualizations were performed using GraphPad Prism 10.0 and MS Excel software.

## Figures and Tables

**Table 1:** Particle size, polydispersity index, and zeta potentials of liposomal formulations.

Formulation Code	Particle Size (nm) ± SD	PDI ± SD	Zeta Potential (mV) ± SD
L1	185.32 ± 0.80	0.206 ± 0.012	35.0 ± 0.5
L2	219.90 ± 2.05	0.251 ± 0.018	38.2 ± 0.8
L3	208.72 ± 3.09	0.212 ± 0.023	2.2 ± 0.8

**Table 2:** Zone inhibition diameter (mm) values of postbiotic lysate and postbiotic-containing liposomal gel formulation against various strains.

	Microorganisms				
	*P. aeruginosa*	*S. aureus*	*E. coli*	*Enterococcus hirae*	*C. albicans*
Ofloxacin	21	23	16	12	-
Nystatin	-	-	-	-	20
Postbiotic	9	10	9	10	8
Liposomal gel with postbiotics	9	9	10	8	7

**Table 3:** MIC (%) values of postbiotic lysate and postbiotic-containing liposomal gel formulations against various strains.

	Microorganisms			
	*P. aeruginosa*	*S. aureus*	*E. coli*	*C. albicans*
Postbiotic	3.125%	12.5%	12.5%	3.125%
Liposomal gel with postbiotics	1.25%	5%	2.5%	1.25%

**Table 4:** Formulations and time to reach the critical TSI value (hours).

TSI	L1	L2	L3
3	01:30:00	00:45:00	01:30:00
10	05:15:00	02:45:00	02:45:00

**Table 5:** Time-dependent particle size, polydispersity index, and zeta potential values of the formulation at 4 ± 2 °C (60 ± 5% RH) (n = 6).

Code	Time (Day)	Particle Size (nm) ± SD	PDI ± SD	Zeta Potential (mV) ± SD
L1	0	185.32 ± 0.80	0.205 ± 0.012	35.0 ± 0.5
	15	183.02 ± 1.41	0.213 ± 0.011	34.9 ± 1.6
	30	187.58 ± 0.82	0.211 ± 0.008	32.7 ± 1.7
	60	187.87 ± 1.41	0.226 ± 0.005	32.5 ± 2.6
	90	204.38 ± 2.07	0.222 ± 0.015	28.9 ± 1.9
	180	274.88 ± 1.84	0.304 ± 0.021	28.5 ± 1.1

**Table 6:** Time-dependent particle size, polydispersity index, and zeta potential values of the formulation at 25 ± 2 °C (60 ± 5% RH) (n = 6).

Code	Time (Day)	Particle Size (nm) ± SD	PDI ± SD	Zeta Potential (mV) ± SD
L1	0	185.32 ± 0.80	0.205 ± 0.012	35.0 ± 0.5
	15	219.90 ± 2.05	0.251 ± 0.018	31.3 ± 1.2
	30	329.12 ± 4.93	0.372 ± 0.051	28.9 ± 3.5
	60	981.55 ± 80.10	0.615 ± 0.025	20.9 ± 1.6
	90	1878.33 ± 120.55	>1	14.9 ± 3.6
	180	4337.00 ± 1736.86	>1	13.6 ± 3.2

**Table 7:** Ingredients and codes of liposome formulations.

Formulation Code	Ingredients	Molar Ratio
L1	H100:DOTAP:CHOL	60:10:30
L2	P100:DOTAP:CHOL	60:10:30
L3	P100-3:DOTAP:CHOL	60:10:30

**Table 8:** Ingredients of liposome–gel formulations.

Ingredients	%
Postbiotic-containing liposome	5
Propylene glycol	10
Glycerin	2
Antimicrobial Preservative	0.95
Gelling agent	0.5
Triethanolamine (TEA)	0.45
Ultrapure water	81.1

**Table 9:** Liposome–gel formulations and codes.

Formulation Code	Ingredients
LG1	U-21 (0.5%) Gel + L1
LG2	U-21 (0.5%) Gel + L2
LG3	U-21 (0.5%) Gel + L3
LG4	U-30 (0.5%) Gel + L1
LG5	U-30 (0.5%) Gel + L2
LG6	U-30 (0.5%) Gel + L3

**Table 10:** Mathematical equations of the kinetic models.

Kinetic Model	Equation
Zero-order	Q = k_0_t
First-order	ln(1 − Q) = −k_1_t
Higuchi	Q = k_h_t^1/2^
Korsmeyer–Peppas	Q = kt^n^
Hixson–Crowell	Q^1/3^ − W_t_ = k_hc_t

## Data Availability

The original contributions presented in this study are included in the article. Further inquiries can be directed to the corresponding author.
